# Progress in Pediatric Asthma Surveillance II: Geospatial Patterns of Asthma in Alameda County, California

**Published:** 2006-06-15

**Authors:** Paul B English, Eric M Roberts, Michelle Wong, Craig Wolff, Samuel Valdez, Stephen K Van den Eeden, G. Thomas Ray

**Affiliations:** California Department of Health Services, Environmental Health Investigations Branch; California Department of Health Services, Environmental Health Investigations Branch, Richmond, Calif; California Department of Health Services, Environmental Health Investigations Branch, Richmond, Calif; California Department of Health Services, Environmental Health Investigations Branch, Richmond, Calif; California Department of Health Services, Environmental Health Investigations Branch, Richmond, Calif; Kaiser Permanente of Northern California, Division of Research, Oakland, Calif; Kaiser Permanente of Northern California, Division of Research, Oakland, Calif

## Abstract

**Introduction:**

As with many diseases, the epidemic of asthma among children over the past few decades has been shaped by a social and environmental context that is becoming progressively more evident. Commonly used methods for asthma surveillance, however, are based on national rather than local data. The purpose of this study was to develop high-resolution asthma surveillance techniques responsive to the needs of health care professionals and local child health and social justice advocates.

**Methods:**

We assembled a working data set of health care use records from 2001 from public and private sources covering 1.7 million person-months among children younger than 18 years in Alameda County, California. Health care use was categorized by type and analyzed by census tract demographic information. Images of the geographic distribution of health service events were created using density estimation mapping with overlapping 0.5-mile (805-m) radius spatial buffers, and statistical significance (two-tailed *P* < .05) was estimated using a Monte Carlo simulation algorithm.

**Results:**

High-poverty communities had higher rates of emergency department visits due to asthma than low-poverty communities but had lower rates for indicators of quality primary asthma care. Geospatial analysis enabled visualization of this phenomenon; it further detected areas with elevated emergency department visit rates and potentially related environmental hazards in and around communities of concern. Areas of the county not previously considered to be deeply burdened by asthma were identified as having high emergency department visit rates.

**Conclusion:**

The assembly and high-resolution geospatial analysis of health care use data contributed to a more detailed depiction of pediatric asthma disparities than was previously available to community members, public health professionals, and clinicians. Information generated using these techniques facilitated discussion among stakeholders of the environmental and social contexts of asthma and health disparities in general. Proceedings of group evaluations suggested that the material aided in the translation of data describing spatial variations in health event risk to address specific community experiences and concerns.

## Introduction

### Tools for asthma surveillance

As with many diseases, the epidemic of asthma among children over the past few decades has been shaped by social and environmental factors that are becoming progressively evident. The disproportionate burden of the disease borne by low-income communities and communities of color has long been noted (1-3). Increasing evidence that the disease and its consequences are influenced by both access to health care (4-6) and environmental factors (7-10) has contributed to the formulation of asthma as a social and environmental justice issue (11,12). As communities mobilize resources to address asthma, considerations such as economic parity, the built environment, segregation, housing quality, and local sources of pollution have become at least as prominent as considerations related to the clinical management of the disease (13-17).

Commonly used methods for asthma surveillance, however, are based on national rather than local data. Much of our knowledge about asthma prevalence and disparities is based on nationwide surveys such as the National Health Interview Survey, the National Health and Nutrition Examination Survey, the National Ambulatory Care Survey, and the Behavioral Risk Factor Surveillance System (18). Counties and organizations for which local data are important, however, must frequently rely on inference when applying these general findings to their communities (19,20).

The cost and complexity of population-based health surveys places such activities beyond the reach of most communities (21,22), so they generally rely on hospitalization rates due to asthma to quantify the problem in their areas. Because of the large sample sizes required to calculate such rates, data must generally be aggregated over multiple years and under the best of circumstances are likely to be available only at the level of the postal ZIP code (23-25). Although these data are helpful, they are of limited use for advocates seeking to target limited resources and generate awareness of specific environmental determinants such as roadways, ports, or industrial facilities for the following reasons (26):

- Hospitalization rates reflect only the most severe or poorly controlled asthma.
- Communities of concern are often smaller than ZIP codes or cross ZIP-code boundaries and require higher geographic resolution for health surveillance.
- When traditional mapping techniques are used, gradations of health risk are misrepresented as abrupt changes at ZIP-code boundaries.
- Large, thinly populated areas, although lacking in valid data because of the instability of calculated rates, convey the greatest visual impact.

As part of the California Environmental Health Tracking Program (CEHTP), we sought to develop techniques for disseminating asthma surveillance data that are responsive to community needs. Databases were assembled containing geocodable information describing health care use related to asthma, a process described in the accompanying paper (27). Here we describe the analysis of this database in addition to the results and their potential contributions to local efforts to prevent and treat asthma.

### Asthma in Alameda County, California

Alameda County is a mostly urban county in the San Francisco Bay area of northern California, with a population of approximately 1.4 million. Diverse socioeconomic strata and ethnic groups are represented in the county, and 27.1% of residents were born outside of the United States. Of these immigrants, approximately half are from Asia, and almost one third are from Latin America.

**Figure 1:**
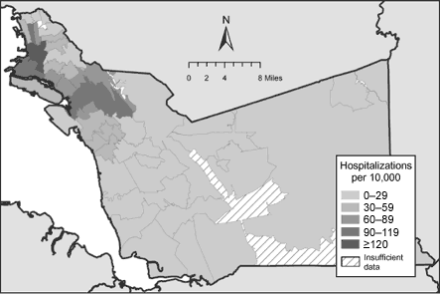
Asthma-related hospitalization rates among children aged 0 to 14 years in Alameda County, California, by ZIP-code tabulation area (ZCTA) of residence, 1998–2000 (23).

Among children, Alameda County's hospitalization rate due to asthma is the second highest in the state (384 per 100,000 children aged 14 years and younger) (23,28). Hospitalization rates are commonly cited to describe the problem of asthma in the county because they are the only metric available at the ZIP-code level. As shown in Figure 1, an area beginning along the San Francisco Bay in the northwestern part of the county and stretching eastward across the city of Oakland is commonly noted to include the communities of greatest concern regarding asthma burden. One ZIP code in the neighborhood of West Oakland, 94607, has been noted to have a pediatric asthma hospitalization rate seven times the statewide average (28). This area is perceived to bear a disproportionate burden of air pollution compared with others in the county due to the proximity of many interstate highways, heavy industry, and the presence of a major Pacific coast shipping port.

### Community needs for asthma surveillance

To complement the development of asthma surveillance techniques, CEHTP initiated a stakeholder input process to learn about the needs and uses for Alameda County asthma data and to incorporate the principles and components of community-based participatory research (29). In all, representatives from more than 20 local organizations participated, such as those from community groups, city and county health agencies, health and environmental advocacy groups, environmental and social justice groups, health care providers, and legislative staffers from a local city council. Using this stakeholder input, we have been able to characterize some of the current asthma information needs and activities in Alameda County.

Generally, stakeholders use or would like to use asthma information to plan, target, or obtain specific resources such as funding, outreach and education activities, case identification and management, health services, and pollution-reduction efforts. Information about asthma is also used for advocacy, decision making, and evaluation of policies on issues such as public health, the environment, and urban planning. Finally, some stakeholders use asthma data to initiate discussion and to organize efforts among their communities as well as to confirm what is suspected or already known about asthma at the community level.

Accordingly, stakeholders characterized useful asthma information as community-level information for multiple asthma indicators. Asthma data that have been analyzed, interpreted, or presented in a social, economic, or environmental context (e.g., analyzed with relation to race or ethnicity, income, accessibility of health care, air quality) are also considered useful. Asthma data should be easily comparable among communities and with county, state, and national data. Lastly, asthma information should be reliable, available in a timely and regularly updated fashion, and presented in an accessible and comprehensible format (30).

## Methods

### Data sources

The sources and methods for data processing for this project, in addition to discussion of data quality and representativeness, have been described in a companion paper (27). In brief, all Alameda County, California, patient records for enrollees younger than 18 years during 2001 in Kaiser Permanente of Northern California (n = 135,380, geocoding success rate 94%) and Medi-Cal (the California Medicaid program) fee-for-service (n = 41,409, geocoding success rate 90%) were assembled and geocoded. Together, these data represented approximately 1.7 million person-months of health care use. Analysis demonstrated that a range of socioeconomic strata was represented in the data set. Hospitalization records from the data set were noted to poorly reflect known spatial variation in asthma hospitalization risk; records describing other health events, however, appeared to correlate well with external data sets and demonstrated high internal consistency. Although it was a nonrandom sample of the county population, this data set contained information on approximately one of every two child residents (or one in three, if calculated in person-months).

### Variables

Outcome variables were chosen to include both severe or poorly controlled asthma and less-severe, or well-controlled, asthma. Previous analysis (27) and clinical reasoning suggested that emergency department visits, outpatient visits, symptom medication purchases, and maintenance medication purchases constituted indicators of a range of disease. Emergency department visits were considered indicators of poorly controlled asthma; outpatient visits, symptom medication purchases, and maintenance medication purchases were considered indicators of well-controlled asthma.

Emergency department and outpatient visits were defined as asthma related if the primary *International Classification of Diseases, Ninth Revision, Clinical Modification* (*ICD-9-CM*) diagnosis code began with the digits 493. For records that included a secondary diagnosis code, events were included if the secondary diagnosis code began with 493 and the primary diagnosis was a condition commonly precipitated by an asthma exacerbation such as pneumonia or respiratory failure. To ensure that multiple billing entries arising from a single visit were not counted as multiple events, it was assumed that a single person could only make one outpatient or emergency department visit on any given date.

Records of medication purchases were not commonly recorded with an *ICD-9-CM* diagnosis. Medications were considered *symptom*, or rescue, medications if they were long- or short-acting beta agonists or anticholinergics. *Maintenance*, or controller, medications included inhaled corticosteroids, methylxanthines, mast cell stabilizers, and antileukotrienes. Because oral corticosteroids are sometimes taken for conditions other than asthma, these medications were excluded to maximize the specificity of this indicator.

### Disparity estimation

To compare the frequency of health care use among communities of different socioeconomic status, we coded each resident address as belonging to a high-poverty (20% percent or more of households) or low-poverty (less than 20% of households) census tract according to the definition employed by the U.S. Census Bureau (31).

Denominators were the numbers of person-months each child was eligible for health care in the census tract category in question; rates were converted to events per person-year for statistical presentation. To allow for the magnification of the standard errors of the estimates from some individuals having multiple asthma-related health care use events, we calculated confidence intervals using a method adapted from Carriere and Roos (32).

### Density estimation mapping

**Figure 2:**
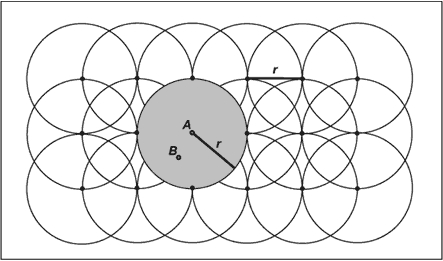
Density estimation method for asthma-related health care use. Distance *r* is set to 0.5 miles (805 m). Rates for grid points (e.g., *A*) are calculated for the populations residing within buffers of radius *r*. To generate continuous, or raster surfaces, nongrid points (e.g., *B*) are assigned inverse-distance weighted averages of their nearest eight neighboring grid point rates. Data were visualized following the procedure of Rushton and Lolonis (26).

Data were mapped following the procedure of Rushton and Lolonis (26). In brief, a grid of regularly spaced points was calculated for the entire county at 0.5-mile intervals. Overlapping buffers were designated as circles of 0.5-mile radii around each point in the grid (Figure 2). In an effort to minimize representation of unstable rates in thinly populated areas of the county, buffers were included in the analysis if they overlaid enough addresses so that the expected frequency for a given event was four or greater. This criterion resulted in a minimum number of residents ranging from 475 addresses per buffer for the least common event (emergency department visits) to 15 addresses per buffer for the most common event (symptom medication purchasing). The number of buffers used depended on the event. Between 863 and 1195 buffers were included in the analyses.

Continuous, or raster, surfaces were generated using an inverse distance weighting algorithm that considered the values of the nearest eight buffer centroids. Surfaces were represented by six color gradations; for consistency, cutoff values between gradations were chosen such that the color representing the highest rates included the 95th percentile rate and above for the map. Colors representing lower rates were chosen using an equal-distance algorithm ranging from the 95th percentile to zero.

### Monte Carlo simulation

The methods for calculating the statistical significance of buffer rates were influenced by the fact that spatial autocorrelation and the overlapping of buffers violated assumptions of independence of measures. Therefore, standard tests of statistical significance assuming independence and normality of distributions could not be used. Monte Carlo simulation was performed by calculating 1000 sets of events and assuming a uniform spatial distribution for the given set of 176,789 addresses and 1.7 million person-months. Statistically, buffers were considered significantly different from the countywide mean if they were below the 2.5th percentile or above the 97.5th percentile for this distribution (the equivalent of a two-tailed significance test with α = .05).

### Community review

We reviewed the raster and Monte Carlo simulation images in formal meetings with stakeholders to assess their impressions of communities highlighted by the findings and to determine whether the images corresponded with their local experiences. We also superimposed these images on municipal maps and used them to select the census tracts that the areas of concern roughly overlay. In this way, some basic demographic data about the highlighted areas could be assembled.

### Software

Residence addresses were standardized using ZP4 (Semaphore Corp, Pismo Beach, Calif) and were subsequently geocoded using a custom application written in Java 2 Platform, Standard Edition (Sun Microsystems Inc, Santa Clara, Calif) and ArcSDE version 9.0 (ESRI, Redlands, Calif). Geocoded address coordinates were taken from the first successful match of the following four street centerline data sets (in order): GDT Dynamap/2000 version 13 (Tele Atlas, Lebanon, NH), NavStreets (Navteq, Chicago, Ill), Tele Atlas MultiNet (Tele Atlas, Lebanon, NH), and the Census 2000 TIGER/Line (U.S. Census Bureau, Washington, DC). For each street centerline data set, the first attempt to match an address was made by indexing the address's ZIP code. If the first match attempt failed, the Soundex phonetic code of the address's city was matched against an index of the Soundex phonetic code of the street centerline's post office name, based on its ZIP code. Applications for creating Soundex indices were written in Java 2 Platform, Standard Edition, using standard methods (33).

Custom applications for assigning geocoded addresses to grid buffers were written using ArcObjects version 9.0 (ESRI, Redlands, Calif). Monte Carlo simulations were performed by applications written in Java 2 Platform, Standard Edition, and Transact-SQL (Microsoft, Corp, Redmond, Wash) and analyzed using SAS version 8.02 (SAS Institute, Cary, NC). Maps were generated using ArcMap version 9.0 (ESRI, Redlands, Calif).

## Results

### Socioeconomic disparities

The rates per 1000 person-years are shown in Table 1. As expected, residents in high-poverty census tracts had rates of emergency department visits due to asthma approximately 66% higher than those in more affluent census tracts. This relationship was reversed for indicators of relatively well-controlled asthma; the reverse disparity became more pronounced for each indicator of higher-quality care. For maintenance medications, the purchasing rate was approximately 41% lower among high-poverty census tract residents than among low-poverty census tract residents.

### Geographic patterns

**Figure 3:**
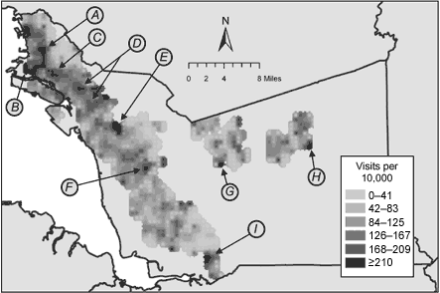
Raster surface for rates of asthma-related emergency department visits among Kaiser Permanente and Medi-Cal fee-for-service enrollees younger than 18 years in 2001, Alameda County, California. Subsets of the total areas having statistically significantly elevated rates are indicated with arrows. (A, neighborhood of North Oakland; B, neighborhood of West Oakland; C, neighborhood of San Antonio; D, neighborhood of East Oakland; E, western portion of city of Castro Valley; F, neighborhood of South Hayward; G, southwestern portion of city of Pleasanton; H, southeastern portion of city of Livermore; I, southwestern portion of city of Fremont.)

**Figure 4:**
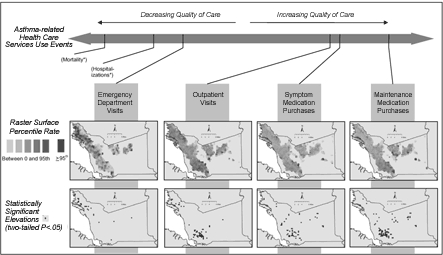
Raster surfaces and statistically significant elevations in the spectrum of asthma-related health care use among Kaiser Permanente and Medi-Cal fee-for-service enrollees younger than 18 years in 2001, Alameda County, California. (Asterisks indicate measures that are not available in the data set.)

Raster surfaces and the geographic distribution of statistically significant elevations for each health care use rate are shown in Figures 3 and 4. The distribution of emergency department visits due to asthma (Figure 3) is very similar to that of hospitalizations depicted using Office of Statewide Healthcare Planning and Development data (Figure 1); the difference was that density estimation produced a higher resolution map. Within ZIP codes known to have elevated asthma hospitalization rates, areas of concern became visible. Also, additional discrete areas with elevated health care use rates became apparent in several ZIP codes in the far southern and eastern regions of the county.

Review of the raster surfaces and Monte Carlo simulations with stakeholders revealed that many of the areas with statistically significant elevations of emergency department visit rates were recognized by participants as having substantial social and health problems and corresponded with community perceptions of asthma burden. Some elevated areas fit the expected profile of high-poverty neighborhoods, which included a majority of residents of color, and some did not (Table 2).

As predicted by the inconsistent disparities in asthma-related health care use represented in Table 1, the geographic distribution of events shifts as indicators of severe or poorly controlled asthma progress to those of milder or well-controlled asthma (Figure 4). For the most extreme indicator of well-controlled asthma — the purchasing of maintenance medications — the northwestern portions of the county are noticeably devoid of rate elevations, whereas southern and eastern portions reveal the largest concentration of such elevations.

## Discussion

These methods were developed for ongoing, inexpensive asthma surveillance that would be relevant to organizations (both governmental and community based) with interests in overall health, environmental health, and social justice. The potential for sustained asthma surveillance of this kind in additional locations is discussed in a companion paper (27). The experiences we report suggest that sophisticated tools for the visualization of asthma disparities in combination with tabular and conventional methods can be instrumental in promoting dialogue among stakeholders.

### Communities of particular concern

We selected some of the areas noted to have statistically significant elevations in rates of asthma-related emergency department visits, an indicator we considered to correspond with more or poorly controlled asthma, for further analysis (Figure 3 and Table 2). From the ZIP-code–level analyses and investigations from the county Public Health Department (34), some of these communities (A through D) had previously been noted as having serious concerns about asthma, although previous data had not allowed for high-resolution mapping; these were also neighborhoods that local stakeholders had identified during the course of their work as being of particular concern. Others (E through I), however, had not previously been highlighted in this way.

Previous investigators (18) have reported that the burden of asthma falls most heavily on low-income communities and communities of color, and the overall patterns noted in Table 1 are consistent with these reports. In spite of this finding, the areas highlighted by the estimation density mapping show striking heterogeneity. Some neighborhoods, such as East Oakland and West Oakland, are home to community groups with histories of advocacy work surrounding environmental justice issues. These communities are widely thought to experience a disproportionate exposure to diesel truck emissions, industrial sources of air toxins, waste disposal sites, and the Port of Oakland, a major Pacific Ocean shipping hub. Almost all are proximal to major interstate highways (other than the area in Livermore), although some in particular either include major nexuses of multiple highways (West Oakland and North Oakland) or directly border interstate 880 (including San Antonio, East Oakland, South Hayward, and South Fremont), which has some of the most truck traffic in the county. Based on analysis of the census tracts roughly overlying these communities, an array of racial or ethnic groups and socioeconomic strata are represented (Table 2).

### Enhanced asthma surveillance

Among the advantages of our procedures was increased mapping resolution, which allowed observation of geographic variations that could be directly related to local concerns by stakeholders with knowledge of neighborhood demographics, resource needs, and sources of pollution that had been identified as priorities of interest by community residents. Through collaboration with local government, nongovernmental, and community-based organizations, we were able to assess the usefulness of these techniques for addressing stakeholder concerns. For example, one community representative noted that the emergency department raster surface vividly conveyed that many in her community rely heavily on local emergency departments as sources of primary care. Other stakeholders were able to use the maps to delineate communities affected by their proximity to multiple interstate roadways and lack of health and social services. Similarly, discussion of thinly populated areas of the county for which substantive data were not available was minimized.

The maps also enabled us to discover new information about the problem of asthma in Alameda County. First, they refined our understanding that, although the areas with high asthma in the northwestern portions of the county may be due in part to environment and housing conditions in that area, some of the elevations in hospitalization and emergency department visit rates there are clearly associated with lower use of primary care services, such as outpatient visits and maintenance medication purchases. Primary care use has been shown to prevent emergency department visits, hospitalizations, and deaths due to asthma (4-6,35-39).  

Second, the visualization of primary care indicators of asthma (outpatient visits, symptom medication purchases, and maintenance medication purchases) revealed areas in the southern and eastern portions of the county with elevated rates that had not previously been discussed as part of the asthma problem in the county among local public health agencies, the state Department of Health Services, or local nongovernmental or community-based organizations. Not only did visualization of primary care indicators of asthma provide new information but it also stimulated dialogue about what constitutes a public health "problem" in the county. Although asthma may be more endemic to these areas than previously recognized, it seems to be well-controlled and not associated with some of the morbidity observed elsewhere in the county. On the other hand, it still may constitute a problem from a primary prevention perspective or to the extent that it indicates that ambient environmental conditions in these locations are adversely affecting the health of residents.

### Usefulness for stakeholders

We thought it was important that surveillance data be useful to a range of constituents. The ability to convey disparities in asthma burden at the community level by using multiple indicators had been identified by our stakeholder group as particularly valuable. Although stakeholders so far have had limited opportunities to use these data, they have been able to describe several potential uses for this type of health surveillance. In particular, countywide providers discussed strategies to address geographic differences in health service accessibility and the need to increase communication among health care providers. Community advocates were able to further inform discussions about health resource issues, as well as concerns about local pollution sources.

Stakeholders also prioritized having asthma data that could be compared with state and national statistics, but this was an area in which our analysis fell short. The data are drawn from a nonrandom sample of Alameda County residents. The nonrandom aspect of the sample discourages generalization to the county level and subsequent comparison to external standard populations.

None of the four population measures of asthma burden (emergency department visits, outpatient visits, symptom medication purchases, and maintenance medication purchases) represent true asthma prevalence because they are all confounded by social factors such as access to care (40). We found this confounding to be both an advantage and a disadvantage. On one hand, we would have liked to have produced a single map that directly answered the question, "Where is the asthma in Alameda County?" On the other hand, we found that stakeholders expected indicators of asthma prevalence to be inextricably bound with social structural factors such as access to care or social stressors (41), and they demonstrated a capacity for using tools that incorporated those interrelated factors.

### Limitations

Our use of a nonrandom sample is the foremost limitation of our data. In recognition of this limitation, we chose not to calculate neighborhood or countywide rates of health care use for comparison with known rates calculated from random, national-level samples. Instead we focused on geographic comparisons within the county. Although such comparisons may still be subject to sample bias, they are less problematic than comparisons to external rates calculated using differing methods. As noted elsewhere (27), a high proportion of county residents are represented in the sample, and the geographic distribution of emergency department visits is highly correlated with the distribution known for asthma-related hospitalizations. Therefore, our focus on intracounty geographic variations in events rates appears reasonable.

For calculations of statistical significance, our methods do not avoid repeated measures. Given our selected significance level (α = .05), 5% of all buffer centroids (between 40 and 60 centroids per map) should be expected to have rates significantly different from the county average. Therefore, it is important to realize that this technique is not appropriate for testing for the existence of within-county disparities in health care use rates; it can, however, delineate patterns in disparities that are presumed to exist. In light of extensive evidence documenting the existence of social disparities in asthma, the assumption of the existence of such disparities in Alameda County seems valid. Furthermore, statistically significant elevations that appear in clusters, as many of those found in this project do, are less likely to be products of random variation.

Address-geocoded coordinates are only as good as the street centerline software used in their calculation. The primary product that we used (GDT Dynamap/2000) was updated by the manufacturer before this project began, but there remain errors in the coding of geographic coordinates over which we have little control. As we incorporate such technologies into ongoing surveillance efforts, we expect systematic analysis of error rates relative to a sampling of true geopositioned ground coordinates may be useful. 

Finally, diagnostic imprecision is a limitation of all epidemiological studies of asthma, and our study is no exception. Attribution of emergency department and outpatient visits to asthma was based on billing codes, a method which, even when accurate, may artificially simplify complex diagnostic situations. We based our classification of long-acting beta agonists as symptom medications on their relatively low efficacy in preventing hospitalizations (42), but reasonable people could argue that these should be considered maintenance medications. Similarly, we excluded oral corticosteroids from our analysis based on concerns that their use is not sufficiently specific to asthma. Again, it could be argued that inclusion of these medications would increase the sensitivity of the indicator in a way that would justify the loss of specificity.

### Conclusions

For this project, we sought to develop asthma surveillance techniques using 1) large health service data sets covering various asthma indicators, 2) sophisticated GIS methodologies, and 3) ongoing public stakeholder dialogue. Increases in mapping resolution seem to represent a substantial improvement in the information that has been available historically, and portions of the study area have been newly identified as being potentially endemic to asthma. We also have found we are able to present a more nuanced picture of asthma in our study area that incorporated aspects of both the physical environment (e.g., traffic pollution) and the social environment (e.g., health care access). Although this information is only now being introduced, preliminary discussions suggest that this information is responsive to the needs of local government, nongovernmental, and community-based stakeholder needs.

## Figures and Tables

**Table 1:** Rates of Asthma-related Health Care Use Among Kaiser Permanente of Northern California and Fee-for-Service Medi-Cal Enrollees Younger Than 18 Years by Poverty Rate Census Tract, Alameda County, California, 2001

**Health Care Use Event**	**Low Poverty^a^ (95% CI)^b^**	**High Poverty^a^ (95% CI) b**
Emergency department visit	7.0 (6.4-7.6)	11.6 (9.9-13.2)
Outpatient visit	155 (151-159)	141 (132-150)
Symptom medication purchase	264 (258-270)	215 (203-226)
Maintenance medication purchase	158 (153-162)	94 (87-101)

a aAll rates are per 1000 person-years. Low poverty is defined as less than 20% of households meeting the poverty threshold; high poverty is defined as 20% or more of households meeting the poverty threshold (31).

b Confidence intervals (CIs) were calculated using a method adapted from Carriere and Roos (32).

**Table 2:** Descriptions of Selected Alameda County, California Areas With Elevated Rates^a^ of Asthma-related Emergency Department Visits

**Area**	**Corresponding Reference Letter for Figure 3**	**Predominant (>50%) Racial or Ethnic Group**	**Poverty Rate, %**	**Asthma Previously a Concern^b^**
Neighborhood of North Oakland	A	African American	29.6	Yes
Neighborhood of West Oakland	B	African American	34.5	Yes
Neighborhood of San Antonio	C	NA	25.7	Yes
Neighborhood of East Oakland	D	NA	25.5	Yes
Western portion of city of Castro Valley	E	White	6.1	No
Neighborhood of South Hayward	F	NA	7.2	No
Southwestern portion of city of Pleasanton	G	White	3.3	No
Southeastern portion of city of Livermore	H	White	5.4	No
Southern portion of city of Fremont	I	Asian	2.9	No

NA indicates not applicable — areas for which no single racial or ethnic group predominates.

a Statistical significance (two-tailed *P* < .05) was estimated using a Monte Carlo simulation algorithm. The source for these rate calculations is Kaiser Permanente and fee-for-service Medi-Cal administrative records.

b Yes indicates that the community had previously been mentioned as a concern in discussions about asthma with county agencies (32).
