# Supplementary material for: Progress in Pediatric Asthma Surveillance II: Geospatial Patterns of Asthma in Alameda County, California
Source: Prev Chronic Dis. 2006 Jun 15;3(3):A92. (PMC1637800)

Figure 4. Raster surfaces and statistically significant elevations in the spectrum of asthma-related health care use among Kaiser Permanente and Medi-Cal fee-for-service enrollees younger than 18 in 2001, Alameda County, California. (Asterisks indicate measures that are not available in the data set.)

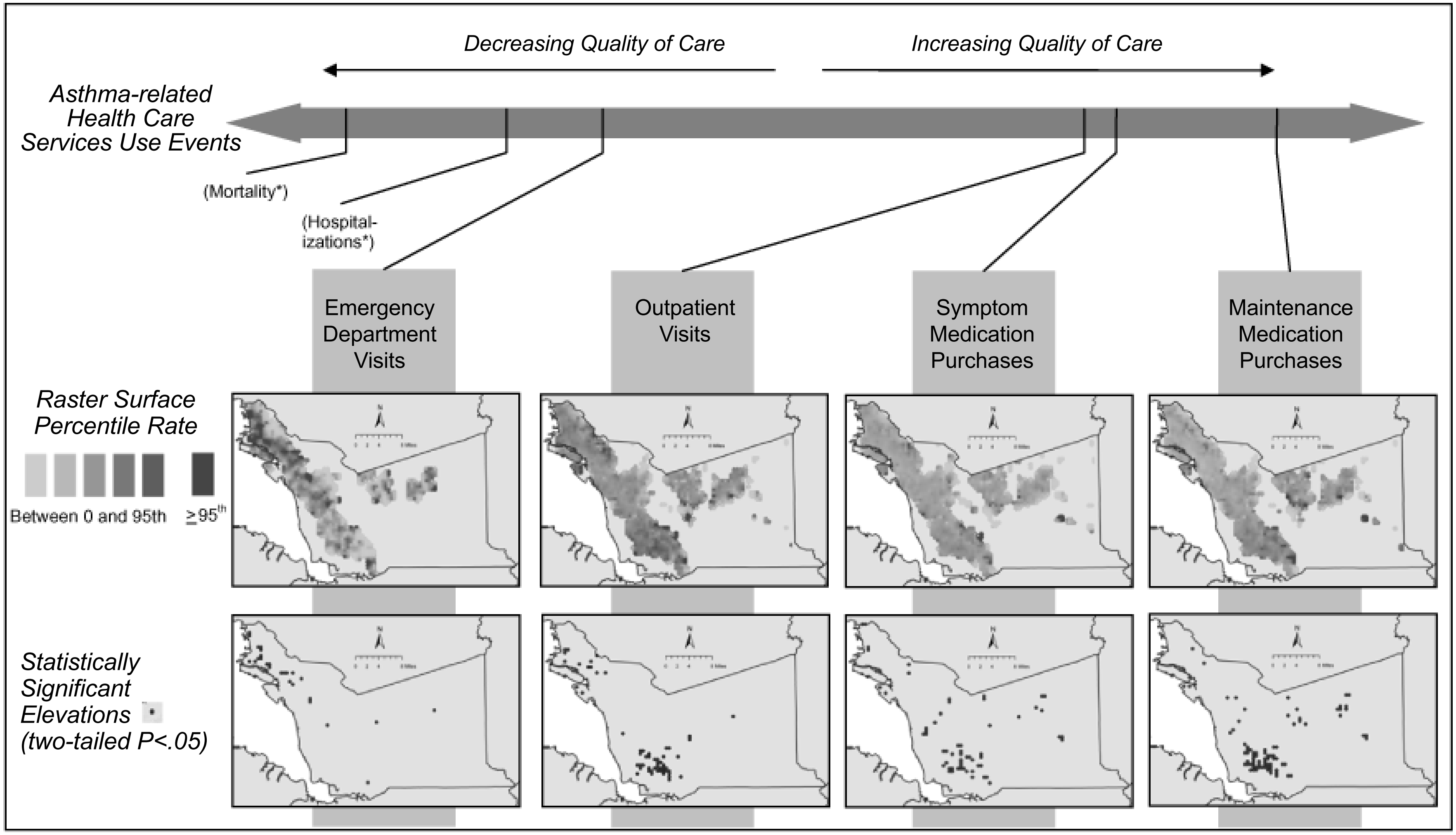

Supplement: Supplementary file 1 [file 05_0187_01.pdf]
